# The Snoring Index Identifies Risk of Non-Alcoholic Fatty Liver Disease in Patients with Obstructive Sleep Apnea Syndrome

**DOI:** 10.3390/biology11010010

**Published:** 2021-12-22

**Authors:** Katharina Bahr, Perikles Simon, Barbara Leggewie, Haralampos Gouveris, Jörn Schattenberg

**Affiliations:** 1 Department of Otorhinolaryngology, Head and Neck Surgery, University Medical Center Mainz, 55131 Mainz, Germany; Barbara.Leggewie@ukbonn.de (B.L.); haralampos.gouveris@unimedizin-mainz.de (H.G.); 2 Department of Sports Medicine, Johannes-Gutenberg-University, 55122 Mainz, Germany; simonpe@uni-mainz.de; 3 Department of Otorhinolaryngology, Head and Neck Surgery, University Hospital Bonn, 53127 Bonn, Germany; 4 Metabolic Liver Research Program, I. Department of Medicine, University Medical Center Mainz, 55131 Mainz, Germany; Joern.Schattenberg@unimedizin-mainz.de

**Keywords:** obstructive sleep apnea, non-alcoholic fatty liver disease, AHI, snoring, CAP, transient elastography

## Abstract

**Simple Summary:**

Obstructive sleep apnea syndrome (OSA) and non-alcoholic fatty liver disease (NAFLD) are very common and share underlying metabolic risk factors. It remains unclear how exactly the two diseases are related. In this study, patients with obstructive sleep apnea were evaluated for the presence of NAFLD with the aim of finding parameters in polysomnography that may indicate NAFLD. The snoring index turned out to be the most valuable and a very reliable screening tool for the presence of NAFLD—independent of other metabolic risk factors.

**Abstract:**

Background: The aim of this observational cohort study was to explore the severity of liver disease in patients with suspected obstructive sleep apnea in Germany. Methods: Patients undergoing polysomnography or home sleep apnea testing (HSAT) as an evaluation for the presence of OSA were screened using vibration-controlled transient elastography (VCTE) and continuous attenuation parameter (CAP) with a Fibroscan ® Mini 430. Clinical and laboratory data were collected following the overnight exam. Results: In total, 78 patients (28 female (35.9%), mean age 54.2 years) with OSA defined by an apnea-hypopnea-index >5 events/hour were included between OCT 2020 and APR 2021. Patients exhibited a high metabolic risk profile with 17% known diabetes mellitus type 2 (T2D), 62% arterial hypertension, 14% hyperlipidemia and 36% BMI > 30 kg/m^2^. The prevalence of steatosis defined by a CAP > 280 dB/m was 54%. The prevalence of at least significant fibrosis was 16% (E > 9.0 kPa). Interestingly, patients with a snoring index above the median of 278/h showed significantly higher CAP-values (*p* = 0.0002). In addition, the proportion of oxygen saturations below 90% (t90) correlated with CAP-values (*p* = 0.02), as well as metabolic risk factors including increased waist circumference (*p* = 0.005) and body mass index (BMI) (*p* = 0.035). On the other hand, the apnea-hypopnea-index (AHI) as a marker of OSA severity did not correlate with VCTE, CAP or laboratory parameters. Conclusion: Patients with moderate to severe OSA have a high prevalence of hepatic steatosis. The snoring index is an easy-to-use clinical tool to identify patients at risk for relevant liver disease within the larger group of patients with OSA.

## 1. Introduction

It is estimated that 23.4% of women and 49.7% of men suffer from moderate to severe obstructive sleep apnea (OSA) in central Europe [1]. Like OSA, the non-alcoholic fatty liver (NAFL) shows a high prevalence in the general population. The term NAFL comprises different histologically defined stages—including non-alcoholic fatty liver disease (NAFLD) and steatohepatitis (NASH), up to hepatocellular carcinoma (HCC). The pathophysiology is currently under intense exploration. The role of a metabolic–inflammatory phenotype is emerging as the common ground and driver for extrahepatic diseases [2]. Obstructive sleep apnea, characterized by nightly interruptions of breathing during sleep and sleep disruption due to arousals [3] often associated with loud snoring, is believed to promote the development of NAFL [4,5]. Even mild OSA and snoring without OSA seem to be associated with systemic inflammation as well as local inflammation, e.g., chronic nasal inflammation, in otherwise healthy individuals [6,7]. OSA is characterized by cyclic intermittent nocturnal hypoxia as a consequence of recurrent apneas and hypopneas. OSA has various effects on the organism. Nocturnal hypoxia was shown to be significantly associated with oxidative stress, an increase in pro-inflammatory markers as well as an imbalance in nitric oxide production and damage to the endothelium [8]. Intermittent hypoxia has also shown to cause insulin resistance, and dysfunction of key steps in hepatic lipid metabolism, atherosclerosis, hepatic steatosis and fibrosis, each of which is pertinent to the development and/or progression of NAFLD [9]. 

NAFL may be clinically inapparent and is considered reversible, or it may become noticeable with progression to NAFLD through non-specific symptoms such as fatigue, feeling of fullness, feeling of pressure or tenderness in the right upper abdomen. Transition from NAFLD to NASH occurs about 10–15% of the time. In patients with NASH, fibrosis develops in a quarter of the patients; 2–5% of them develop cirrhosis within one year. This in turn may lead to hepatocellular carcinoma (HCC) with a probability of 2–3% [10]. A prevalence of 20–30% for NAFLD is reported in Europe and the Middle East [11,12]. It is even expected that the number of deaths caused by NAFLD and secondary diseases will continue to rise until the year 2030 [11]. The increase in NAFLD cases is most likely due to demographic changes as well as the higher prevalence of lifestyle-associated risk factors caused by hypercaloric diet and lack of exercise, leading to the metabolic syndrome. Three most common causes of increased morbidity and mortality associated with NAFLD are cardiovascular disease, liver disease, and cancer. Further, there remains an unmet need for safe and effective therapies on these extra- and intrahepatic manifestations among patients with NAFLD [13]. 

The comorbidities of both OSA and NAFL include metabolic syndrome, obesity, arteriosclerosis, hypertension and increased inflammation [14,15,16,17]. Previous studies dealing with NAFLD and OSA are, for example, a meta-analysis by Jin et al. [18] from the year 2018. It included nine studies with a total number of patients of 2272, and stated that OSA was significantly correlated with steatosis, detected on liver biopsy. It also found that the severity of OSA was associated with higher levels of alanine transaminase (ALT), while age, gender, the presence of diabetes and body mass index did not show an association with ALT levels [18]. 

Despite the current knowledge on the principal association of OSA with potentially the second hit of NAFL, little is known on the pathophysiological role of OSA for the progression of NAFL or vice versa and current explanations remain hypothetical. On the one hand, periods of decreased oxygen saturation due to OSA may accelerate inflammatory processes in NAFL due to ischemia re-oxygenation. On the other hand, the progression of NAFL and its association with inflammation, metabolic disease and intra-abdominal mechanical irritation of breathing in NAFL may be stressful in supine position. With our contribution here, we aim at elucidating the OSA-specific pathophysiological effects associated with NAFL.

Clarification of the aspect whether it is rather OSA causing progression in NAFL or NAFL causing OSA, would clearly benefit from investigating the main pathophysiological aspects of OSA with NAFL. 

We aimed to screen patients with OSA for the presence of steatosis and fibrosis in order to study the effects of OSA parameters with NAFL and to identify the sleep parameters that are actually linked to NAFL.

## 2. Materials and Methods

In this observational cohort study patients with confirmed OSA who presented at the sleep outpatient department between OCT 2020 and APR 2021 were included in the study and screened for NAFLD via liver elastography. In order to exclude alcoholic fatty liver, women had to consume less than 10 g of alcohol and men less than 20 g per day. Apart from regular alcohol intake, other exclusion criteria were age <18 years and previously treated OSA as well as previously diagnosed NAFL and other hepatic disease. OSA diagnosis had to be drawn from either home sleep apnea testing (HSAT)/polygraphy or inpatient polysomnography (PSG). If both HSAT and PSG were performed, PSG recordings, known to be more accurate than HSAT, were included in the analysis. If two consecutive nights of PSG had been performed, the second night was included in analysis to avoid a possible first-night effect [19]. The classification of the degree of respiratory distress in sleep apnea patients was performed according to current established AASM criteria [20,21]. Parameters such as apnea-hypopnea-index, snoring index, desaturation index, time spent below oxygen saturation of 90% as well as leg-movement indices, were drawn from the reports. For NAFLD assessment, after anamnesis for alcohol intake and other metabolic comorbidities such as hypertension and diabetes, the waist circumference was measured at the midpoint between the lower margin of the last palpable rib and the most cranially palpable part of the iliac crest, using a stretch-resistant tape. In addition, blood analysis was performed including alanine aminotransferase (ALT or GPT), aspartate aminotransferase (AST or GOT) and platelet count. Next step was the vibration-controlled transient elastography (VCTE) via upper abdominal sonography. VCTE of the liver was carried out with the FibroScan^®^ 430 mini device from Echosens, Waltham, MA, USA. The transducer is placed at liver level in an intercostal space. The head contains a 50-Hertz vibrator that emits a low-frequency wave with a propagation velocity of 1 m/s. 

A singular, mechanical pulse is emitted in the direction of the liver. A 5-MHz ultrasound probe integrated in the probe head measures the propagation speed of the pulse wave in the liver tissue. The stiffer or more inelastic the liver tissue, the faster the pulse travels through the liver and the higher the measured values. The velocity of propagation is proportional to liver stiffness and is expressed as a numerical value with the unit kilo-Pascal (kPa). A healthy liver measures 3–4 kPa. An advantage of the examination is the independence from the examiner. However, the examiner needs experience in finding the optimal point of examination. At least 10 ultrasound waves were emitted and evaluated per examination. A median was calculated by the device. Each patient’s examination took about 5 min.

**Figure 1:**
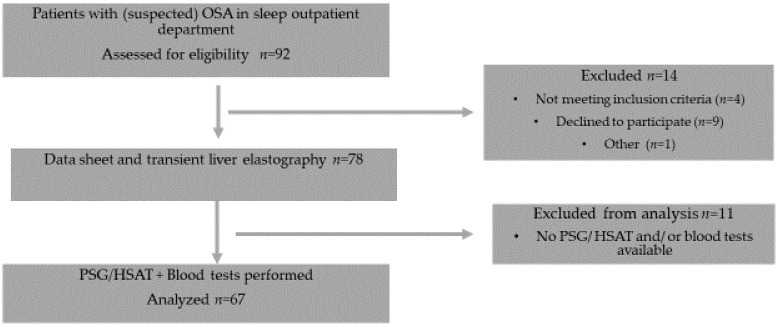
Consort flow diagram of the study protocol.

The assessment of hepatic steatosis was performed by measuring controlled attenuation parameter (CAP), the level of fibrosis was estimated with the liver stiffness measurement (LSM) value. Figure 1 shows a consort flow diagram of the study protocol. 

**Figure 2:**
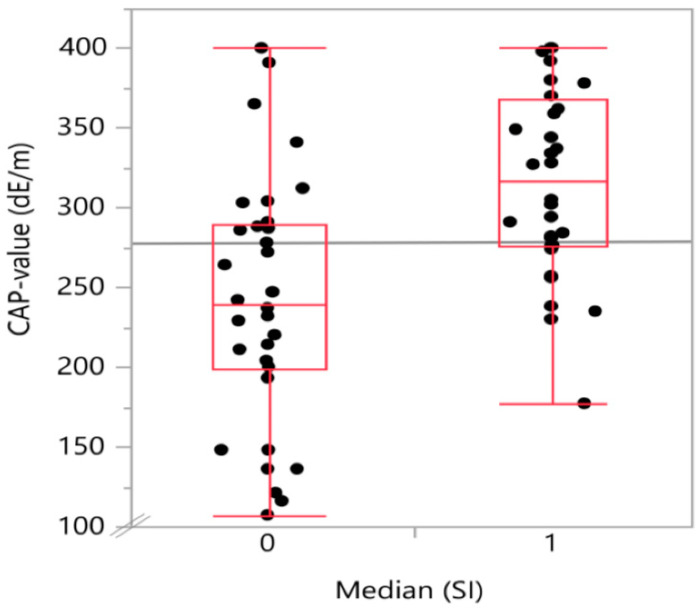
Boxplot with the distribution of CAP-value (continuous attenuation parameter-value) on transient elastography in the two snoring index groups divided into above (1) or below (0) median SI (snoring index). The group of patients with above median snoring index showed significantly higher CAP-values.

**Figure 3:**
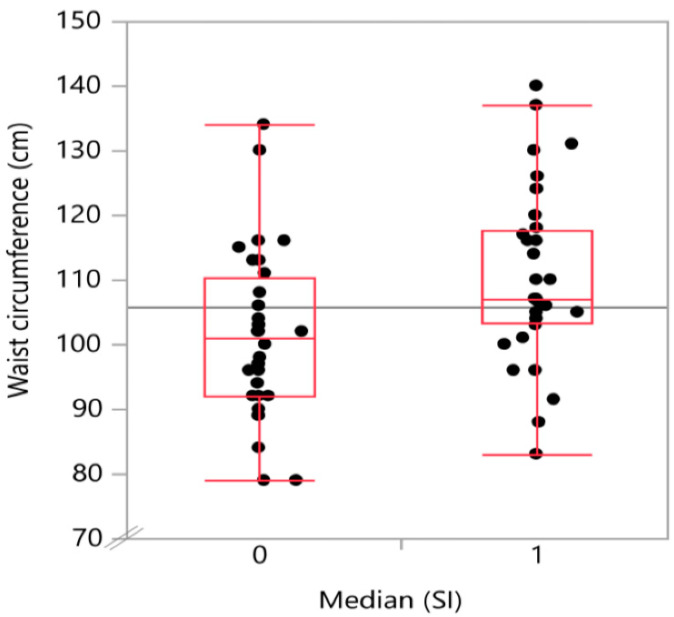
Boxplot with the distribution of waist circumference by snoring index groups. A higher snoring index was associated with a higher waist circumference.

The data assessed (Table 1) were not normal distributed as indicated by Shapiro–Wilk Test and therefore correlation analysis was carried out using Spearman’s correlations (Table 2). Due to the high number of correlations, *p*-values were corrected using Bonferroni–Holm correction for multiple comparisons. For further analysis of the association of the snoring index (SI) with the CAP-values and the waist circumference, we dichotomized the SI-values by a median split and analyzed the difference between below and above median by Wilcoxon test, showing boxplots with median, and 75 and 90% percentiles displaying raw data points (Figure 2 and Figure 3). We employed step-wise logistic regression analysis to assess the combined effects of several variables on the CAP-values and to control for potential confounding variables such as age and gender. Since both SI and CAP-values showed signs of non-normality, we used Box–Cox Transformation to assess the best transformation. This analysis revealed that the CAP-values should rather remain untransformed, and the SI-values should be normalized by the third root. BMI data had to be log-normalized. False discovery rate (FDR) adjusted p-values for the influencing factors on CAP-values were reported.

The study was conducted according to the ethical guidelines of the Helsinki Declaration of 1975 (sixth revision, 2008). Patients gave informed consent for participation in the study. The study was registered, and the Ethics Committee of the State Medical Association of Rhineland-Palatinate (Nr 2021-15648) approved the protocol.

## 3. Results

Of the 78 patients that were found suitable, 67 patients (28 female, 38%) underwent the complete examinations. The patients were on average 54.2 (range 25–83) years old, and the mean BMI was 29.9 kg/m^2^ (range 19.7–49.3 kg/m^2^). Median AHI was 34.3/h (range 4.5/h–122/h). According to the classification of the severity of OSA, 15 patients had mild OSA with an AHI of 5 to <15/h, 19 patients had moderate OSA, AHI of 15 to < 30/h, and 32 patients had severe obstructive sleep apnea with an AHI ≥ 30/h. Patients snored on average 265 times per hour (range 0–1031/h). The average oxygen saturation SpO2, calculated over the total sleep time (TST), was 93.4% on average (range 87–100%). The oxygen desaturation index, ODI, was 32.3/h on average. The range was from 3.9 to 131/h. Percentage of TST for oxygen saturation being below 90% (t90) was on average 8.5% (range 0–73%). The mean CAP-value on TE was 280 (range 107–400), LSM-value was on average 8.1 kPa (range 1.8–70.9kPa), GOT was 31.4 U/L (range 27–65 U/L), and GPT 33 U/L (range 16–65 U/L).

The mentioned parameters and demographic data of the participants are shown in Table 1.

In transient elastography, valid LSM values could be determined in 77 patients. A minimum LSM value of 2.7 kPa and a maximum of 70.9 kPa were measured. The mean value was 8.1 kPa. Out of 77 patients, 12 showed an LSM-value above the threshold of 9 kPa. We consider LSM > 9 kPa to be at least intermediate fibrosis and LSM > 12 kPa suggestive of cirrhosis [22], likely post-NASH. Two patients had LSM-values that were highly indicative of liver cirrhosis (values of 70.9 and 59 kPa).

Six patients had an elevated ALT (2 males, 4 females) and nine patients an elevated AST (8 female, 1 male) value in the laboratory analysis (limit value for women 35 U/L and men 50 U/L).

In summary, out of the 77 participants, none of whom knew of any liver involvement prior to their participation to the study, 41 showed signs of hepatic steatosis, 12 of hepatic fibrosis, and 2 of them even of severe fibrosis (cirrhosis).

The degree of steatosis, determined by semi-quantitative sonographic recording, showed a strong positive (Spearman’s) correlation with the snoring index (Spearman’s ρ = 0.52; *p* < 0.001) as displayed in Table 2. There was also a positive, significant correlation of t90 with both waist circumference (Spearman’s ρ = 0.46; *p* < 0.001) and body mass index (Spearman’s ρ = 0.392; *p* = 0.0035). A graphical representation on how the CAP-values and the waist circumference values are distributed in the below (0) and above (1) median SI groups is given in Figure 2 and Figure 3, respectively.

In the single factorial analysis of CAP value by above (1) or below (0) median [SI], the Wilcoxon/Kruskal–Wallis test (rank sums) showed a p-value for the comparison of the two groups of 0.0002. The same analysis for waist circumference divided between the groups by above (1) or below (0) median (SI) showed a p-value of 0.0072.

The “probability > chi^2^” provides the *p*-value for the group comparison across the two different snoring groups for the “analyzed parameter” CAP-value (“probability” > chi^2^ = 0.002) and waist circumference (“probability > chi^2^” = 0.0071), respectively.

In contrast to t90, median oxygen saturation and ODI correlated neither with the LSM nor with the CAP-value. Interestingly, there was a significant correlation between median oxygen saturation and ALT (Spearman’s ρ = −0.38; *p* = 0.05) as well as platelet count (Spearman’s ρ = 0.381; *p* < 0.002). Nonetheless, neither the serum values of ALT or AST, nor the platelet count, correlated with the level of steatosis or the LSM value. All the calculations and Spearman’s values are shown in Table 2.

**Figure 4:**
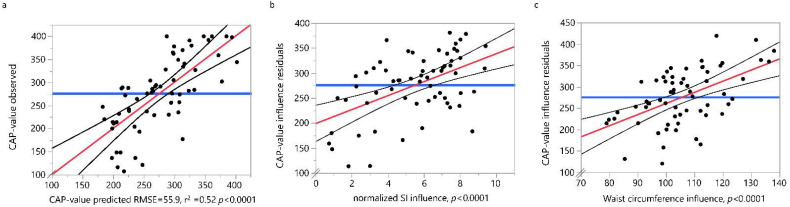
Linear fit of the observed CAP-values by the predicted values (**a**) for the linear regression model with the normalized SI (**b**) and waist circumference (**c**) as influencing factors. Values (black dots), regression line (red line) and 95% confidence intervals for the regression (black curved lines) are shown. RSME = Root Mean Square Error. SI = snoring index, CAP-value = continuous attenuation parameter.

Step-wise logistic regression analysis was used to assess the effects of age, gender, waist circumference, log-normalized BMI and normalized SI on CAP-values. The significance level for entering a single variable into the regression equation was set to 0.05. Only waist circumference and SI emerged as potential predictors for CAP-values and the effects between these two predictors on CAP-values was assessed by linear regression analysis. A significant regression was found F(1, 63, 32.3, *p* < 0.0001) with an R^2^ of 0.52 and with SI (FDR *p* = 0.0002) and waist circumference (FDR *p* = 0.0002) as influencing factors (Figure 4).

In summary, the severity of sleep apnea, classified by AHI, did not correlate with obesity nor any diagnostic tool to classify NAFL. The snoring index emerged as a valid predictor of NAFLD on TE.

## 4. Discussion

This study showed that the severity of respiratory distress in obstructive sleep apnea, represented by the AHI, does not correlate with the degree of steatosis on TE and with ALT levels, although a majority of the patients with OSA in our cohort had a significant level of steatosis on liver TE examination.

Strengths of this study are the availability of data on various stages of obstructive sleep apnea. All patients were naïve to OSA and NAFLD therapy. The association between snoring index and diagnosed steatosis is very strong (*p* < 0.001).

Another strength of our study is the quantification of snoring. Many studies that investigate the influence of snoring used self-reporting of snoring. In the past, this has occasionally turned out to be rather inaccurate, since women tend to under-report and men tend to over-report the snoring activity at night [23,24].

A limitation of this study is that established scores on liver fibrosis could not be calculated since no extensive laboratory chemical analyses were performed as part of routine clinical practice.

Another limitation of the study is the lack of a control group, since only OSA patients were included. A study by Cakmak et al. from the year 2015 managed to find OSA and non-OSA patients, who showed all levels of steatosis on examination, including Level 0 (no NASH). The authors were able to perform group comparisons between different stages of NAFLD and found that oxygen desaturation index values and AHI were significantly higher in moderate and severe NAFLD compared to mild and non-NAFLD groups. In addition, the lowest oxygen saturation values were found in mild, moderate and severe NAFLD compared to the non-NAFLD group in a statistically significant manner [25]. In this study, ALT levels were also higher in severe OSA compared to milder and non-OSA groups. Unfortunately, there was no analysis of fibrosis level in this article.

A case-control study by Singh et al. identified diverse risk factors of NAFLD [26]. NAFL and NASH occur in the setting of peripheral insulin resistance. OSA has been proposed as an independent risk factor for insulin resistance [27]. Whereas metabolic diseases such as diabetes and hypertension, and symptoms such as snoring were considered in our study, there is lack of lifestyle and dietary facts. According to Singh et al., Indian patients with a non-vegetarian diet, lack of regular exercise, and/or intake of fried and/or spicy food, have a higher prevalence of NAFL.

Previously published studies on this topic often only refer to the laboratory value ALT. Even if ALT-values are associated with the AHI, NASH on the other hand could only be diagnosed or excluded with poor accuracy [28]. Patients may have advanced NAFLD, metabolically conditioned, although their ALT values do not show any pathological increase [29]. These studies support our findings in the present study, since the majority of ALT-values of our participants were normal. The significance of an increase in ALT in metabolically-caused fatty liver disease cannot be attested to a good predictive power with regard to NAFL or fibrosis, and thus a diagnosis based on these laboratory values cannot be valid. Nevertheless, there was a correlation of ALT to the median oxygen saturation in the patients examined here. Even if no steatosis or fibrosis diagnoses were found based on ALT, the result can nevertheless express that hepatocytes react to low nightly oxygen levels with increased disturbances of cell integrity.

We excluded participants with diabetes and alcohol consumption in order to exclude possible confounders for NASH. Retrospectively, drugs with a potential for liver damage could have been excluded, too, in order to be even more precise on analysis. In our study, three patients had co-medications with hepatic metabolism. More precisely, these were phenprocoumon, venlafaxin and tamoxifen, which could have acted as confounders on laboratory values.

Although our inclusion criteria in the beginning of the study aimed for moderate and severe OSA patients, we had twelve patients who turned out to have a mild OSA. This was caused by the AHI on HSAT, which lead to the inclusion and the liver diagnostic. However, the patients received an inpatient polysomnography (PSG) afterwards, in which their AHI turned out to be in the mild range (<15/h). Since PSG is more accurate than HSAT and for consistency purposes, we used the AHI-value of PSG for analysis.

This study emphasized the important role of nocturnal hypoxia levels in the genesis of NAFLD, since an increase of nocturnal hypoxia severity leads to an increase in NAFLD-severity.

If chronic intermittent hypoxia correlates independently with the development and progression of NAFLD, then CPAP or APAP therapy should be an obvious choice. A very detailed paper on this approach was published in 2018 by D. Kim et al. [30]. There, however, NAFL is not investigated, but the transaminases ALT and AST. Here, the authors found a significant improvement of AST and ALT under CPAP therapy. Unfortunately, ALT and AST are not able to detect NAFLD, NASH and fibrosis or document their improvement under APAP/CPAP. Thus, the studies, exclusively showing improvement of liver enzymes, are not suitable to reliably express a positive effect on NASH or fibrosis under CPAP [30,31].

On the contrary, no positive effect of PAP therapy on the course and development of steatosis has yet been proven [32,33]. Further studies on hypoxia-reducing effects on steatosis progression are needed. Recovery of steatosis in these cases, however, should not be measured with laboratory chemical parameters, but rather with sonography and transient elastography.

The association between snoring index and steatosis has been suspected in a few studies before. Wang et al. analyzed data of two ongoing prospective cohort studies with >20,000 participants. They found that snoring was independently and positively associated with higher prevalence and incidence of NAFLD on a ten-year follow-up examination, indicating that habitual snoring is a useful predictor of NAFLD, especially in lean and younger individuals (BMI < 24, age < 45 years). In addition, the association was also higher in individuals with normal triglyceride values and average body waist circumference. Here, in contrast to our study, only the presence of snoring was qualitatively assessed without a snoring index. Patients with confirmed obstructive sleep apnea were excluded, but no confirmation was performed via PSG or HSAT.

Since we are still far from understanding the pathophysiology in the genesis of NASH and NAFLD, explaining the association with comorbidities, such as OSA and snoring, remains difficult. We speculate that a metabolic–inflammatory phenotype, leading to NAFLD and NASH, can be augmented by OSA [2].

Snoring is a strong—maybe even the strongest—indicator of the presence of OSA. However, it can also be present in non-OSA individuals. A study by Sowho et al. indicated that snoring intensity could be an indicator of whether snoring is OSA-associated or not. OSA patients appeared to be more severe snorers in terms of frequency and loudness [34].

This could lead to the assumption that snoring without OSA might have a different pathophysiology. Since our study lacks a control group, we cannot address the question whether the strong link of NAFL to snoring is independent of OSA and if this link would still increase, decrease or even exist in non-OSA snoring individuals.

The question remains how snoring itself affects the genesis of NAFL. Although nasal disorders do not play a fundament role in OSA genesis, snoring is an essential part of OSA and treatment via nasal surgery has demonstrated better quality of life and compliance to CPAP therapy in OSA patients [35,36]. A study by Muenzel et al. gave proof of nightly noise-induced cardiovascular morbidity [37]. It is possible, however, that morbidity extends beyond cardiovascular effects to metabolic effects such as the genesis of NAFL. The pathogenesis in this case, however, remains unclear.

According to our study and following the recommendations of the few other studies that have been discussed previously, snoring individuals should be screened for the presence of NAFL via ultrasonography.

The role of OSA in the strong link between snoring and NAFL remains unclear. The pathogenesis that connects the presence of snoring with the incidence of NAFL needs further investigation.

## 5. Conclusions

In this study, the prevalence of NASH in OSA patients was not significantly higher than expected in the general population. Interestingly, the presence of signs of steatosis and fibrosis was strongly associated with the presence of snoring (snoring index). This highly significant and strong correlation provides preliminary strong evidence for a recommendation to screen snoring patients for steatosis and fibrosis. In particular, patients with fibrosis should be followed up carefully in order to stop or slow the deterioration and, if necessary, to early detect HCC.

## Figures and Tables

**Table 1:** Demographic data and collected parameters.

Parameter	Mean	Range
Age	54.2 years	25–83 years
BMI	29.9 kg/m^2^	19.7–49.3 kg/m^2^
Waist Circumference	106.4 cm	70–152 cm
AHI	34.3 h	5–122/h
Mean Oxygen Saturation	93.4%	87–100%
ODI	32.3/h	3.9–131/h
t90	8.5%	0–73%
Snoring Index (SI)	265/h	0–1031/h
PLM-Index	28.3/h	1–141/h
LM-Index	41.3/h	2.7–191.3/h
CAP-value	280 dB/m	107–400 dB/m
LSM-value	8.1 kPa	1.8–70.9 kPa
GPT	33 U/L	16–65 U/L
GOT	31.3 U/L	27–65 U/L
Platelet Count	257.9	99–460

BMI: body mass index; AHI: apnea-hypopnea-index; ODI: oxygen desaturation index; PLM-Index: periodic leg-movement index; LM-Index: leg-movement index; CAP-value: continuous attenuation parameter-value; LSM: liver stiffness measurement; GPT: glutamate-pyruvate transaminase; glutamic oxaloacetic transaminase.

**Table 2:** Correlation analysis of sleep- and liver-specific parameters using Spearman and Bonferroni–Holm.

Variable 1	Variable 2	Spearman ρ	Probability > |ρ|	Bonferroni–Holm *p*-Value
AHI	t90	0.695	<0.0001	<0.0001 *
Mean oxygen saturation	t90	−0.640	<0.0001	<0.001 *
SI	CAP-value	0.512	<0.0001	<0.001 *
t90	Waist circumference	0.46	0.0001	0.006 *
AHI	Mean oxygen saturation	−0.403	0.0005	0.019 *
Mean oxygen saturation	PLM-Index	−0.41	0.0008	0.028 *
Mean oxygen saturation	LM-Index	−0.405	0.0009	0.032 *
t90	BMI	0.392	0.001	0.036 *
SI	AHI	0.3841	0.001	0.041 *
AHI	CAP-value	0.185	0.121	1.824
AHI	Log [E (kPA)]	0.175	0.141	1.837
AHI	GOT (U/L)	0.003	0.982	0.981
AHI	GPT (U/L)	0.252	0.066	1.191
AHI	Platelet Count	−0.089	0.489	2.926
t90	Platelet Count	−0.071	0.583	2.917
SI	Platelet Count	−0.071	0.587	2.347

SI: snoring index; CAP-value: continuous attenuation parameter-value; t90: percentage of time during the night with blood oxygen saturation below 90%, Log [E]: logarithmic elasticity; AHI: apnea-hypopnea-index; PLM: Periodic Leg Movement; LM-Index: leg-movement index; GOT: glutamic oxaloacetic transaminase or aspartate aminotransferase; GPT: glutamate-pyruvate transaminase or alanine transaminase; BMI: body mass index; * = statistically significant correlation.

## Data Availability

The data presented in this study are available on request from the corresponding author.
